# Generation of Koku-Related Peptides Using Gamma-Glutamyl Transpeptidase Post-Treatment in Porcine Liver Hydrolyzates

**DOI:** 10.3390/ijms27083440

**Published:** 2026-04-11

**Authors:** Manuel Ignacio López-Martínez, Angelina Hopf, Gijs J. C. Vreeke, Fidel Toldrá, Roelant Hilgers, Leticia Mora

**Affiliations:** 1 Laboratory of Food Chemistry, Wageningen University & Research, Bornse Weilanden 9, 6708 WG Wageningen, The Netherlands; mi.lopez@iata.csic.es (M.I.L.-M.);; 2 Instituto de Agroquímica y Tecnología de Alimentos (CSIC), Avenue Agustín Escardino 7, 46980 Paterna, Valencia, Spain

**Keywords:** porcine liver, enzymatic hydrolysis, γ-glutamyl peptides, koku, taste, antioxidant activities, transpeptidase

## Abstract

The growing production volume of the meat industry has increased the need for revalorization of meat by-products to reduce economic and environmental impacts. Enzymatic hydrolysis of protein-rich meat by-products is an effective strategy for producing hydrolyzates with bioactive potential. Combining sequential enzymatic hydrolysis with γ-glutamyl transpeptidase activity can promote the formation of γ-glutamyl peptides associated with koku perception, a sensory attribute that increases taste intensity, continuity, and palatability. This study aimed to develop porcine liver hydrolyzates enriched in koku-related peptides through enzymatic hydrolysis followed by post-treatment with the transpeptidase Protana Uboost. Substrate specificity assays showed that a 0.2 U/mL enzyme concentration maximized γ-glutamyl dipeptide formation. Sequential hydrolysis using Alcalase and Protana Prime followed by Protana Uboost post-treatment generated the highest levels of koku-related peptides. Moreover, post-treatment significantly enhanced antioxidant capacity in the resulting hydrolyzates, supporting their potential as a functional ingredient.

## 1. Introduction

The global meat industry is one of the most productive food sectors, with an annual production exceeding 345 million tons which results in the generation of more than 150 million tons of by-products [1]. The inadequate revalorization of these by-products represents a significant economic loss and exacerbates environmental concerns associated with meat production. Among these edible by-products, edible porcine organs, such as the liver, constitute an underutilized resource despite their high nutritional value, particularly due to their content of proteins with high biological value [2]. Enzymatic protein hydrolysis has emerged as an effective strategy for the revalorization of protein-rich by-products, allowing the generation of hydrolyzates enriched in bioactive peptides with potential health-promoting properties [3]. For industry, the development of antioxidant hydrolyzates can be a promising strategy, as their incorporation into food products may extend shelf life by delaying lipid oxidation. Furthermore, from a human health perspective, antioxidants can help to reduce oxidative stress, thereby protecting cells from damage and contributing to the prevention of chronic diseases [4]. However, a major limitation of protein hydrolyzates as food ingredients is the development of bitterness, mainly attributed to the hydrolytic formation of hydrophobic fragments, which negatively affects their sensory acceptability [5,6,7]. A common strategy to mitigate this problem is the use of sequential enzymatic hydrolysis, where endopeptidases first generate medium-sized peptides, followed by exopeptidases that cleave off terminal hydrophobic residues associated with bitter taste, leading to an overall improvement in taste [7]. Nevertheless, even after sequential hydrolysis, bitter free amino acids may remain in the hydrolyzates, potentially affecting the overall taste profile. To solve this problem, γ-glutamyl transpeptidases (GGTs) have attracted interest due to their ability to catalyze transpeptidation reactions using glutamine as a γ-glutamyl donor and free amino acids as acceptors, resulting in the formation of γ-glutamyl peptides [8]. Mechanistically, γ-glutamyl peptides act as agonists of the calcium-sensing receptor (CaSR), a class C G-protein-coupled receptor located in taste buds and structurally related to sweet and umami receptors. The binding of koku-active γ-glutamyl peptides to CaSR enhances intracellular Ca^2+^ signaling in taste cells and modulates neural pathways associated with basic taste qualities, intensifying the perception of umami, sweet, and salty tastes and contributing to sensory attributes such as mouthfulness, thickness, and continuity [9]. Therefore, the production of γ-glutamyl peptides can be considered a promising strategy to improve both the palatability and functional potential of protein hydrolyzates [10].

The aim of this study was to develop porcine liver hydrolyzates with enhanced antioxidant activity and improved sensory-related properties, focusing on the enrichment of taste-active compounds such as free amino acids and koku-related peptides. Different combinations of proteases were applied to produce the hydrolyzates, which were post-treated with a commercial transpeptidase (Protana Uboost), with or without the addition of free glutamine (20 mmol/L) as a substrate for γ-glutamyl peptide formation. The different conditions tested are compiled in Table 1.

Taste-related compounds including free amino acids and koku-related peptides were analyzed and quantified using (U)HPLC–MS/MS. Finally, the in vitro antioxidant capacity of the hydrolyzates was assessed to determine the potential impact of the transpeptidase post-treatment on their bioactivity.

## 2. Results and Discussion

### 2.1. Enzyme Transpeptidase Activity and Substrate Specificity Assay

The transpeptidase activity of the different proteolytic enzymes (Alcalase: 0.02 ± 0.00 U/mL; Flavourzyme: 0.03 ± 0.00 U/mL; and Protana Prime: 0.01 ± 0.00 U/mL) was evaluated as a control, to ensure that they do not exert transpeptidase activity. In contrast, Protana Uboost presents a high transpeptidase activity (2066.12 ± 210.09 U/mL), as expected. Protana Uboost is known to show γ-glutamyl transferase (GGT) activity, which catalyzes the hydrolysis of Gln into Glu using water as an acceptor substrate for the γ-glutamyl moiety from Gln [11]. However, under transpeptidation conditions, free amino acids can act as acceptors instead of water, resulting in the formation of γ-glutamyl dipeptides.

**Figure 1:**
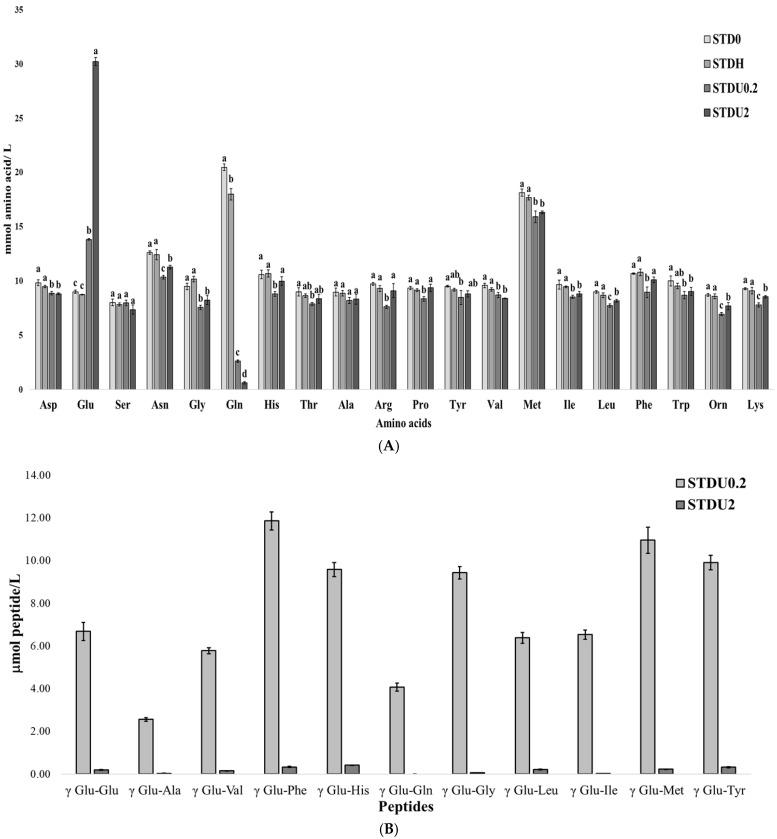
Free amino acids in mmol/L (**A**) and peptides in μmol/L (**B**) quantified in 10 mM free amino acids solution post-treated with Protana Uboost 0.2 U/mL and 2 U/mL. Lowercase different letters (a, b, c) mean significant differences (*p* < 0.05) among all samples.

Figure 1A shows the effect of Protana UBoost activity on free amino acid concentration at different enzyme dosages. Both enzyme concentrations of 0.2 and 2 U/mL exhibited significant decreases (*p* < 0.05) in the concentrations of Asp, Asn, Gly, Gln, Val, Met, Ile, Leu, Trp, Orn, and Lys amino acids compared to the non-supplemented control STD0. Additionally, His, Arg, Pro, Tyr, and Phe were significantly reduced only in STDU0.2. In contrast, higher concentration did not notably affect the amino acid profile. A significant increase (*p* < 0.05) in Glu content was observed in both STDU0.2 and STDU2, suggesting enzymatic conversion of Gln into Glu due to the glutaminase activity of Protana Uboost. In contrast, STDH samples (without the enzyme but following enzymatic conditions) showed a significant reduction only in Gln (*p* < 0.05) in comparison to STD0, suggesting that the temperature, pH and time used for the enzyme treatments were not responsible for the observed decrease in amino acid concentration, with GGT action being solely responsible for this reduction.

The enzyme concentration of Protana Uboost strongly influenced the patterns of amino acid consumption. At an enzyme concentration of 0.2 U/mL, the reductions in Asn, Arg, Pro, Leu, Phe, Orn, and Lys were significantly higher than at 2 U/mL. Although Gln consumption was higher in STDU2, this did not translate into higher γ-glutamyl peptide formation (Figure 1B) but rather increased Glu formation (Figure 1A). This result could be due to glutaminase activity acting as the main activity at high enzyme concentrations (2 U/mL), whereas transpeptidase activity is favored at lower enzyme concentrations (0.2 U/mL).

Regarding individual γ-glutamyl peptides, Figure 1B shows that higher levels of γ-Glu-Phe, γ-Glu-Met, and γ-Glu-Tyr were detected in STDU0.2, whereas γ-Glu-Gln and γ-Glu-Ala were present at lower concentrations, particularly in the 2 U/mL sample (STDU2). STD0 and STDH did not present any detectable γ-glutamyl peptides. This trend is consistent with the amino acid profiles shown in Figure 1A, where Ala did not exhibit a significant reduction (*p* < 0.05) and Gln content is mostly decreased due to its use as a substrate for the generation of γ-glutamyl peptides.

Protana Uboost is a GGT with substrate specificity in the production of γ glutamyl peptides. In this sense, Yang et al. (2018) reported that certain GGTs, such as those from *Bacillus amyloliquefaciens,* preferentially utilize aromatic amino acids, including Phe, Met, and Tyr, as acceptors, whereas Gln and Ala present lower affinity [12]. These substrate preferences are consistent with the results obtained in the present study. Comparable findings evaluating Protana Uboost showed the generation of γ-glutamyl peptides but reported γ-Glu-Gln and γ-Glu-Gly as the most abundant ones [8]. These differences may be due to the use of a shorter reaction time (180 min versus 360 min), higher amino acid concentration (20 mmol/L versus 10 mmol/L), and possible differences in enzyme kinetics under those conditions.

Overall, the results indicate that an enzyme concentration of 0.2 U/mL provides the most favorable balance between transpeptidase and glutaminase activities and, hence, is the most effective to generate γ-glutamyl dipeptides. Consequently, this enzyme-to-substrate ratio was selected for the post-treatment of porcine liver hydrolyzates using the same reaction conditions.

### 2.2. Porcine Liver Characterization

Cooked porcine livers showed values of weight (1668.02 ± 64.15 g), moisture (72.28 ± 0.41 g/100 g), protein (20.85 ± 0.27 g/100 g), pH (6.53 ± 0.03), water activity (0.994 ± 0.000), and color (L* = 48.23 ± 0.66; a* =15.44 ± 0.08; b* = 12.19 ± 0.03, C* = 19.61 ± 0.07, h° = 38.42 ± 0.14) comparable to previous studies [13,14,15]. The nucleotide content (AMP: 3.29 ± 0.03 mg nucleotide/100 g wet matter; IMP: 3.20 ± 0.12 mg nucleotide/100 g wet matter; GMP: 4.05 ± 0.10 mg nucleotide/100 g wet matter) was also comparable with previously reported values in porcine organs [16]. In addition, the taste activity value (TAV) analysis indicated that none of the nucleotides reached a TAV ≥ 1, (AMP: 0.07 ± 0.00; IMP: 0.07 ± 0.00; GMP: 0.32 ± 0.01), suggesting that these compounds do not influence the overall taste of porcine livers. However, previous studies have reported that some 5’-nucleotides, such as AMP, IMP or GMP, can interact synergistically with amino acids like Asp and Glu at umami receptors, increasing umami intensity [17,18]. Although nucleotides do not individually contribute to the overall taste based on TAV, they may be acting as taste modulators.

### 2.3. Analysis of Taste-Related Substances

Sequential hydrolysis was found to increase the degree of hydrolysis (DH) compared to hydrolysis with a single protease, with maximum DH and free amino acid levels obtained with the combination of Alcalase and Protana Prime (HAPP) (Table 2). Unexpectedly, Protana Uboost treatment showed a significant increase in DH (*p* < 0.05) in unhydrolyzed samples (HCU), suggesting that Protana Uboost may exhibit a slight intrinsic hydrolytic activity. Presumably, this is due to their capacity to hydrolyze γ-glutamyl donors, such as endogenous glutamyl peptides or Gln, releasing free Glu that may interact with the OPA reagent [19]. The analysis of free amino acids (FAAs) content revealed a pattern comparable to that observed for DH. Specifically, a significant increase (*p* < 0.05) in all taste-related FAA concentrations (umami, sweet, bittersweet and bitter) and EUC was perceived following this sequence: HC < HA < HAF < HAPP. This is consistent with more intense hydrolysis generally releasing more free amino acids, especially when using Protana Prime as a second enzyme, due to its strong exopeptidase activity [20]. When evaluating the effect of Protana Uboost post-treatment (with no Gln added), a significant reduction (*p* < 0.05) in Gln content was observed in HAU, HAFU, and HAPPU samples. In HAFU and HAPPU, a significant decrease (*p* < 0.05) was observed in Asp, Ser, Gly, Gln, Thr, Lys, Ile, and Phe, and in HAPPU also in Ala, Pro, Val, Cys, His, Tyr, Leu, Asn, and Orn. Such decreases support Gln as a gamma glutamyl donor and free amino acids as acceptors during transpeptidation. Regarding the addition of 20 mmol/L Gln, a similar trend showed an overall decrease in FAAs, although an increase in free Gln (*p* < 0.05) in HCUG, HAUG, HAFUG, and HAPPUG was observed, showing that not all supplemented Gln was consumed in the generation of γ glutamyl peptides. Finally, EUC increased during sequential hydrolysis and remained stable across post-treatments, indicating that umami potential was not reduced by Protana UBoost. These results together with the fact that this post-treatment generates gamma glutamyl peptides with a koku effect suggest that there can be a further enhancement of the overall taste. Table 3 shows that, based on the TAVs, porcine liver hydrolyzates have a taste profile mainly based on umami (Glu) and bittersweet (Val and Lys) flavors, with a slight contribution from sweet (Ala) and bitter (His and Phe) amino acids, especially in HAPP, HAPPU and HAPPUG. Post-treatment with Protana Uboost reduced several taste-active amino acids’ influence, such as Phe or His, due to their utilization as substrates for the formation of γ-glutamyl peptides. Regarding this, a decrease in bitter amino acids can favor the enhancement of other more pleasant tastes (umami, sweet and bittersweet) [21]. In addition, γ-glutamyl peptides could contribute enhancing attributes such as mouthfulness and continuity [8], resulting in a more balanced and palatable taste profile.

Table 4 shows the quantification of peptides associated with potential koku attributes in porcine liver hydrolyzates. Regarding leucyl peptides, Leu-Glu and Leu-Ala concentrations increased significantly (*p* < 0.05) in all hydrolyzates compared to the unhydrolyzed ones, with HAF showing the highest concentration of both. Protana Uboost resulted in a significant increase (*p* < 0.05) in Leu-Ala content in HCU, HCUG, HAU, HAUG, HAPPU and HAPPUG. However, the obtained results suggest that the formation of Leu-Glu and Leu-Ala seems to be mainly influenced by enzymatic hydrolysis and enzyme selection, rather than Protana Uboost post-treatment.

The analysis of the quantified γ-glutamyl dipeptides revealed very low or negligible concentrations for ten of the eleven γ-glutamyl dipeptides in samples without post-treatment (HC, HA, HAF and HAPP), with the only exception being γ-Glu-Val. γ Glu-Val increased significantly (*p* < 0.05) by one-hundred times in HA and HAPP, and two-hundred times in HAF in comparison to HC, suggesting that γ-Glu-Val could be produced by enzymatic hydrolysis of larger γ-glutamyl peptides already present in the liver. On the other hand, Protana Uboost caused a significant increase (*p* < 0.05) in all eleven γ-glutamyl dipeptides. When evaluating the effect of adding 20 mmol/L Gln during the Protana Uboost post-treatment, two different trends were observed. First, in HCUG, a significant increase (*p* < 0.05) in all eleven γ-glutamyl dipeptides was detected, reaching nearly 70-fold higher levels than HC and HCU, and presenting the highest concentrations in γ-Glu-Glu and γ-Glu-Gln content, compared to the other samples. The comparatively high levels of these two peptides are in line with the enhanced Gln concentration in combination with Protana Uboost’s glutaminase activity converting part of Gln to Glu (Figure 1B). This trend is also observed, to a lesser extent, in HAUG samples, with the significant enhancement (*p* < 0.05) of γ-Glu-Glu, in comparison to HA and HAU. Conversely, in the sequential hydrolyzates, a significant decrease (*p* < 0.05) in γ-Glu-Ala, γ-Glu-Val, γ-Glu-Phe, γ-Glu-His, γ-Glu-Leu, γ-Glu-Ile, γ-Glu-Met, and γ-Glu-Tyr was observed in HAFUG and HAPPUG compared to HAFU and HAPPU. In addition, a significant reduction (*p* < 0.05) in γ-Glu-Glu and γ-Glu-Gly was detected exclusively in HAPPUG compared with the other samples. In contrast, γ-Glu-Gln levels increased significantly (*p* < 0.05) in both HAFUG and HAPPUG. These results suggest that the presence of high free Gln concentrations could promote the formation of γ-Glu-Gln and/or larger γ-glutamyl peptides or poly-γ-glutamyl peptides, such as γ-Glu-Glu-Leu which was identified but not quantified. Regarding other γ-glutamyl tripeptides, γ-Glu-Val-Gly was detected at low concentrations in all samples (*p* < 0.05), with significantly higher content in hydrolyzed samples and no extra accumulation upon addition of Protana Uboost. This suggests that primarily enzymatic hydrolysis, rather than transpeptidation, was responsible for its formation. γ-Glu-Cys-Gly was significantly higher in samples without Protana Uboost compared with post-treated samples without Gln, while it was similar when Gln was added. Given that glutathione (γ-Glu-Cys-Gly) is abundant in the liver [22], the decrease after Protana Uboost post-treatment could be attributed to post-treatment conditions (pH variation at 9 and 360 min of thermal treatment at 55 °C) that may induce glutathione degradation. The addition of 20 mmol/L glutamine, with antioxidant activity [23], could protect glutathione levels. Overall, considering the interest in obtaining koku-related peptides, Protana Uboost post-treatment without the addition of 20 mmol/L Gln resulted in the best option for sequential hydrolyzates.

Enzymatic hydrolysis of protein-rich meat by-products represents a promising revalorization strategy because it generates hydrolyzates enriched in bioactive peptides with diverse biological functions. However, proteolysis releases hydrophobic peptides and bitter-tasting amino acids (Tyr, His, Leu, Ile, Phe, and Trp) that can impart pronounced bitterness, compromising palatability [5]. To solve this drawback, several debittering strategies have been developed and, among them, sequential hydrolysis has demonstrated effectiveness. In this approach, an endopeptidase, such as Alcalase, releases larger peptides, which often contributes to bitterness, followed by an exopeptidase, such as Protana Prime, which cleaves hydrophobic terminal residues responsible for bitterness, thereby improving the sensory profile of the hydrolyzate [20]. Similarly, Rezvankhah et al. (2022) reported that sequential hydrolysis using Flavourzyme as a second enzyme preparation can reduce bitterness while enhancing pleasant and meaty tastes [24]. In addition to sequential hydrolysis, post-treatment with γ-glutamyl transferase (GGT) could further reduce bitterness. GGT catalyzes the transfer of γ-glutamyl residues to available acceptor amino acids, leading to the formation of γ-glutamyl peptides while decreasing the bitter-related amino acids content [25]. Several authors reported promising results with the use of GGT in hydrolyzates from porcine raw materials, such as hemoglobin, muscle or plasma [8,25,26], promoting reductions in bitterness and increases in koku sensation by generating several γ-glutamyl peptides. Koku perception is a complex sensory attribute which can increment the perception and duration of desirable tastes such as sweetness, saltiness, and umaminess, as well as modifying other sensory perceptions such as body thickness or continuity, improving the overall palatability of the products [26]. Despite many γ-glutamyl di- and tripeptides showing intrinsic taste at high concentrations (bitter, umami, etc.), they are able to exert a strong koku effect at lower concentrations [22]. Several γ-glutamyl dipeptides (γ-Glu-Glu, γ-Glu-Gly, γ-Glu-Val, γ-Glu-Leu, γ-Glu-His, γ-Glu-Ala, γ-Glu-Gln, γ-Glu-Phe, etc.) and tripeptides (γ-Glu-Cys-Gly and γ-Glu-Val-Gly) present in this study have been suggested to play a key role in the koku perception of parmesan cheese [27] and dry-cured ham [28]. Therefore, it is conceivable that the accumulation of these γ-glutamyl peptides in porcine liver hydrolyzates post-treated with Protana Uboost may exert a similar koku enhancement, resulting in a pleasant overall taste.

γ-glutamyl peptides can act as agonists of the calcium-sensing receptor (CaSR), intensifying the perception of umami, sweet, and salty tastes and contributing to attributes such as mouthfulness, thickness, and continuity [9]. Although γ-glutamyl peptides are primary contributors to koku perception via CaSR, other peptides such as certain leucyl-dipeptides including Leu-Glu or Leu-Ala have also been reported to elicit koku-like effects [29]; however, their mechanisms of action remain less well understood. One of the most compelling aspects of the koku sensation is its ability to enhance umami perception, which itself is characterized by a savory taste and is known to enhance sweetness and saltiness while masking bitterness [30]. Together with the influence on the koku perception of leucyl and gamma glutamyl dipeptides, other substances present in these hydrolyzates can influence an increase in umami perception. In this sense, α-Glu-Glu is reported to be umami [31], and some free amino acids (Glu and Asp) can act synergistically with nucleotides (IMP, AMP and GMP) in umami enhancement [17]. The porcine liver hydrolyzates produced in this study showed EUC values (59.55–113.89 g MSG/100 g) higher than other porcine lung hydrolyzates [32] (21.25–62.85 g MSG/100 g) and comparable to those of umami-rich foods such as soy sauce (46.08–238.11 g MSG/100 g) [33] and broth tablets (85.3 ± 5.73 g MSG/100 g) [34]. Moreover, the matrix effect is important in umami perception; in liquid foods, such as the hydrolyzates of this study, it is reported that the correlation between EUC and sensory analysis may be strong [34]. This fact supports their potential use as functional taste-enhancing ingredients by combining koku and umami effects.

### 2.4. Antioxidant Potential of the Hydrolyzates

**Figure 2:**
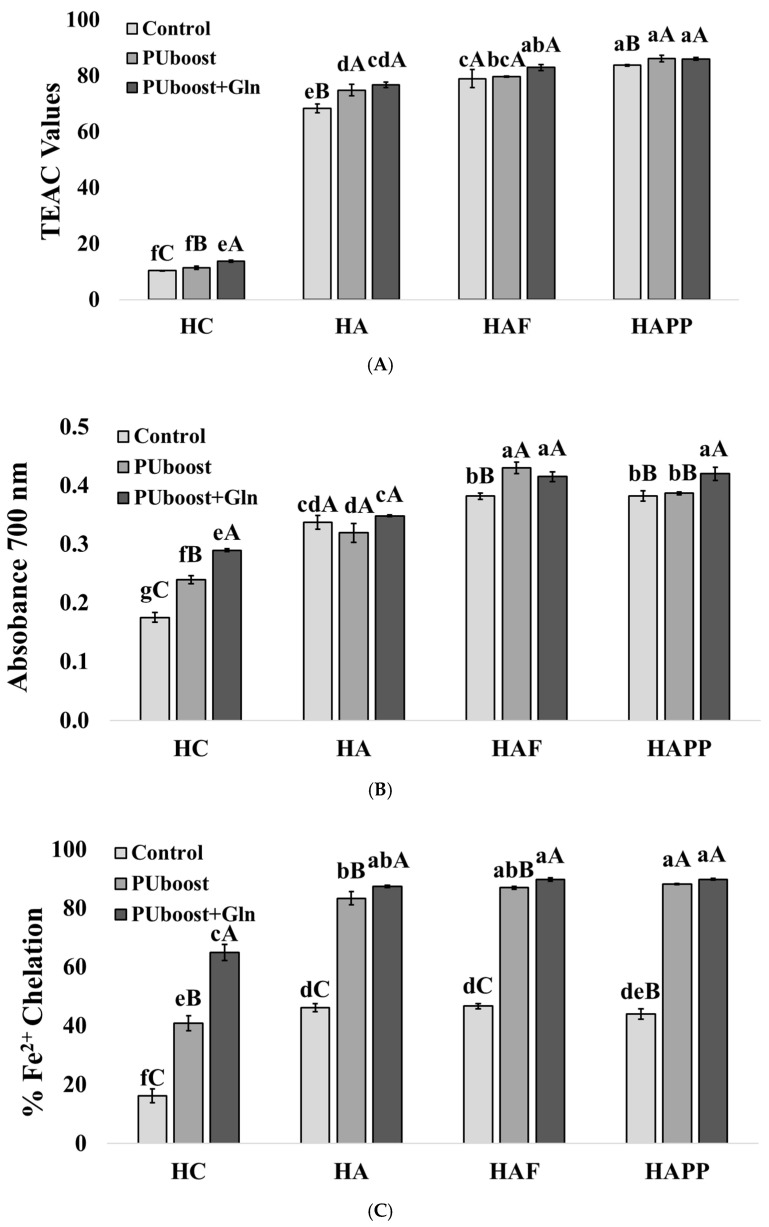

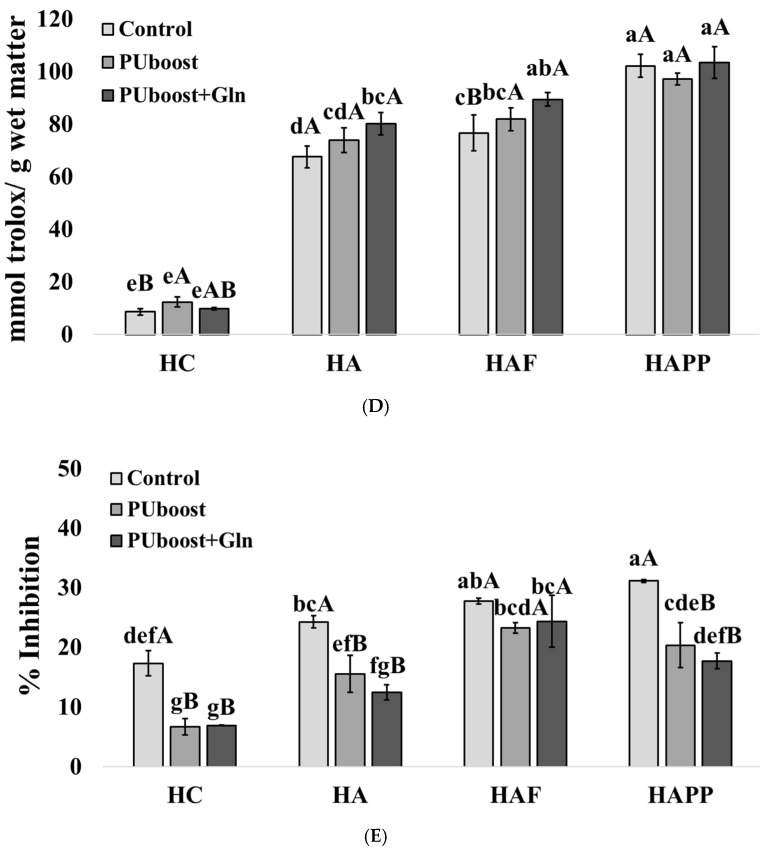
Biological activities and degree of hydrolysis of porcine liver hydrolyzates. (**A**) ABTS assay expressed in TEAC values, (**B**) FRAP assay expressed in absorbance values at 700 nm, (**C**) ferrous chelating activity power expressed as % of chelation, (**D**) ORAC assay expressed in mmol Trolox/g wet matter, and (**E**) DPPH assay expressed in % of DPPH inhibition. Results are expressed as the mean ± SEM of triplicates. Lowercase different letters (a, b, c, d, e, f, and g) mean significant differences (*p* < 0.05) among all samples, while uppercase different letters (A, B, and C) represent significant differences (*p* < 0.05) within the same group (unhydrolyzed samples: HC, HCU, and HCUG; single Alcalase hydrolyzates: HA, HAU, and HAUG; sequential Alcalase–Flavourzyme hydrolyzates: HAF, HAFU, and HAFUG; and sequential Alcalase–Protana Prime hydrolyzates: HAPP, HAPPU, and HAPPUG).

Figure 2 shows a significant increase *(p* < 0.05) in antioxidant activities observed following the sequence HC < HA < HAF < HAPP, indicating that sequential hydrolyzates with the highest degree of hydrolysis showed the highest antioxidant results.

**Figure 3:**
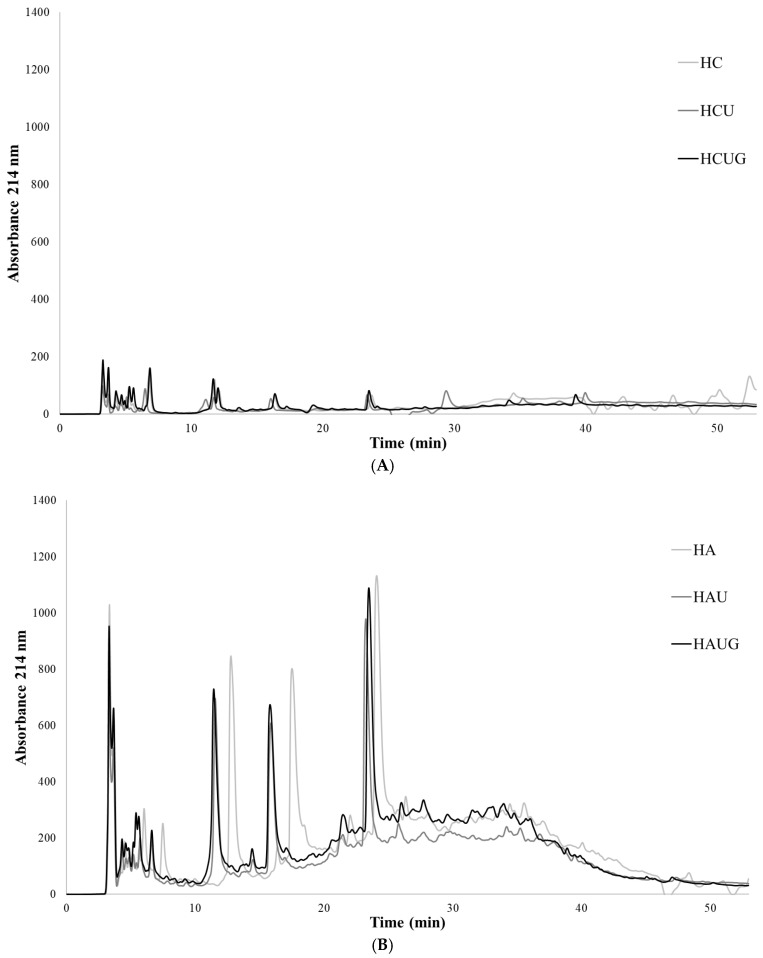

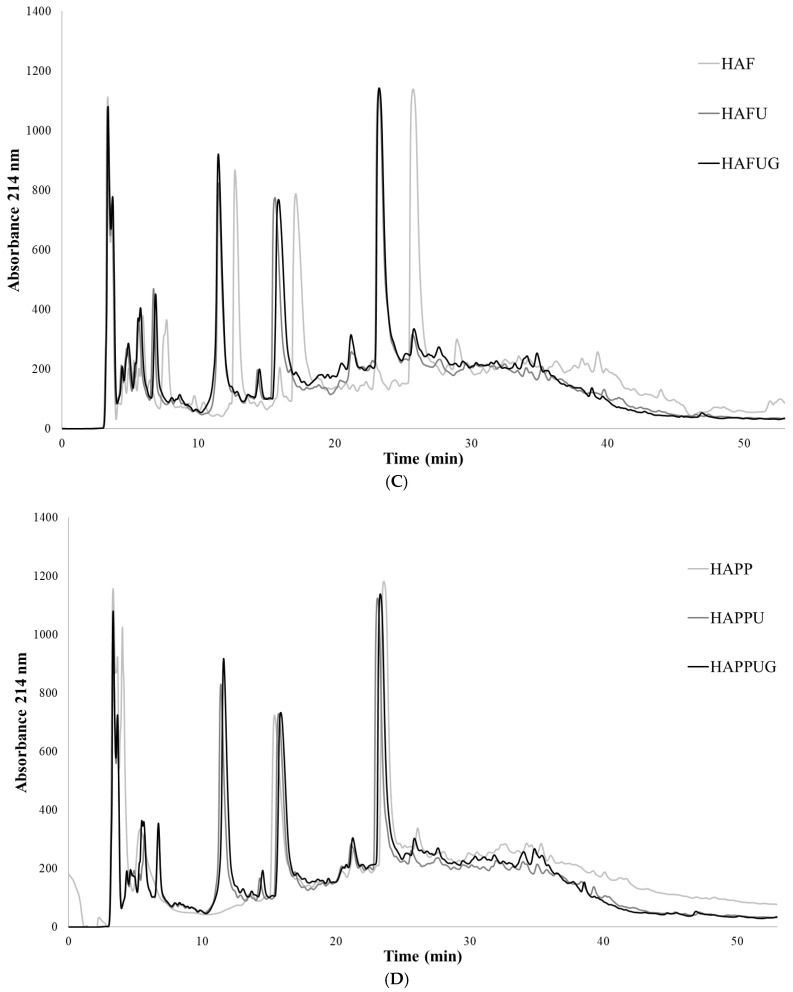
Chromatograms of peptide profile separation according to hydrophobicity for porcine liver hydrolyzates. (**A**) Unhydrolyzed samples; (**B**) Alcalase single hydrolyzates; (**C**) Alcalase–Flavourzyme sequential hydrolyzates; (**D**) Alcalase–Protana Prime sequential hydrolyzates.

Considering the effect of Protana Uboost post-treatment, a significant enhancement (*p* < 0.05) was detected in ABTS (Figure 2A), FRAP (Figure 2B), and Fe^2+^ chelation capacity (Figure 2C) in all sample groups. In addition, ORAC activity (Figure 2D) was significantly increased (*p* < 0.05) in HAUG and HAFUG samples. Regarding the effect of supplementing Gln 20 mM to Protana Uboost post-treatment, a significant increase is observed (*p* < 0.05) in FRAP (Figure 2B) in HCUG, HAFUG and HAPPUG, and in the ferrous ion chelation (Figure 2C) assay in HCUG, HAUG and HAFUG. In contrast, a significant reduction (*p* < 0.05) in DPPH (Figure 2E) radical scavenging activity was observed in all groups post-treated with Protana Uboost. Pearson correlation analysis (Table S2) supported the antioxidant activity results, showing strong positive correlations (ρ > 0.60) between DH and all antioxidant assays, as well as positive correlations between FRAP and ferrous ion chelation with Protana Uboost and Gln addition (ρ > 0.20–0.50), probably due to γ-glutamyl peptide formation and increased free Glu and Gln levels. In contrast, negative correlations were observed between DPPH activity and Protana Uboost or 20 mmol/L Gln addition (ρ < −0.30), indicating a detrimental effect of post-treatments on this antioxidant assay. Sequential protein hydrolysis is well-known to generate low-molecular-weight peptides and free amino acids such as Trp, Tyr [35], His [36] or BCAAs (Leu, Val and Ile) [37] associated with antioxidant activity [3]. In agreement with previous reports, the combined use of Alcalase and Protana Prime has been shown to produce hydrolyzates with high antioxidant capacity from porcine slaughterhouse by-products [20,38]. Furthermore, the formation of γ-glutamyl dipeptides and polypeptides from free amino acid substrates may explain the additional increase in antioxidant activity observed in samples post-treated with Protana Uboost [39]. Peptides containing Glu residues have been reported to promote Fe^3+^ reduction and Fe^2+^ chelation through the action of the carboxyl group [40], which could explain the marked increase in FRAP values and ferrous ion chelating capacity observed in samples treated with Protana Uboost and 20 mmol/L Gln. Free glutamine also shows antioxidant properties by itself [23], further contributing to the observed effects. He et al. (2023) have evaluated the antioxidant and metal-chelating properties of γ-Glu-Trp and γ-Glu-Glu-Trp, reporting that peptides with a higher number of Glu residues exhibited the highest chelating capacity [40]. Several factors influence the antioxidant capacity of protein hydrolyzates, including amino acid composition, molecular weight, hydrophobicity/hydrophilicity, and solubility in the reaction medium, all of which affect peptide–radical interactions in different antioxidant assays. Amino acid residues with aromatic residues (e.g., Phe, Trp), as well as acidic and basic residues (e.g., Glu, His, Lys), have been shown to contribute to radical scavenging and metal-chelating activity, while peptide hydrophobicity and solubility determine their accessibility in specific assay environments such as ABTS, ORAC, or DPPH systems, which differ substantially in solvent compatibility and radical polarity (e.g., ABTS is soluble in both aqueous and organic media, whereas DPPH is largely hydrophobic) and thus differentially reflect hydrophilic versus hydrophobic antioxidant components and peptide interactions [41]. Hydrophilic peptides may show limited interaction with DPPH, and even precipitate due to ethanol-based deproteinization, decreasing the activity [40]. This hypothesis is supported by the peptide profiles shown in Figure 3; shorter retention times were observed in samples HAU, HAUG, HAFU, HAFUG, HAPPU, and HAPPUG. γ-Glutamyl peptides are known to be more hydrophilic than their α-glutamyl peptides, due to the free α-carboxyl group that can interact with water to form hydrogen bonds [42].

Recent studies show that γ-glutamyl peptides such as γ-Glu-Glu-Cys exhibit strong antioxidant properties [43], while others such as γ-Glu-Trp or γ-Glu-Met have been associated with anti-inflammatory [44] and anti-diabetic activities [45]. These findings support the theory that Protana Uboost post-treatment not only improves the koku-enhancing properties of porcine liver protein hydrolyzates but also promotes the generation of peptides with increased bioactive potential, thereby improving their suitability as functional food ingredients.

## 3. Materials and Methods

### 3.1. Chemicals and Reagents

Alcalase 4.0L Pure, Flavourzyme 1000L, Protana Prime and Protana Uboost were obtained from Novozymes (Bagsværd, Denmark). 2,5-Dioxo-1-pyrrolidinyl 6-Quinolylcarbamate 95% (AQC) and Tris (2-carboxyethyl) phosphine HCl (TCEP) were sourced from Activate Scientific (Prien, Germany). Trichloroacetic acid (TCA), formic acid (FA), ferric chloride, potassium ferricyanide, potassium persulfate, sodium hydroxide (NaOH), L-Glutamic acid γ-(p-nitroanilide) hydrochloride (GpNA), Gly-Gly, butylated hydroxytoluene (BHT), 2,2′-azino-bis (3-ethylbenzothiazoline-6-sulfonic acid) (ABTS), 2,2-diphenyl-1-picrylhydrazyl-hydrate (DPPH), o-phthalaldehyde (OPA), sodium dodecyl sulphate (SDS), DL-dithiothreitol (DTT), iron (II) chloride tetrahydrate, sodium 4-[3-(pyridin-2-yl)-6-(4-sulfophenyl)-1,2,4-triazin-5-yl] benzene-1-sulfonate (Ferrozine), 6-hydroxy-2,5,7,8-tetramethylchroman-2-carboxylic acid (Trolox), fluorescein, 2,2′-azobis (2-methylpropionamidine) dihydrochloride (AAPH), perchloric acid, hydrochloric acid, amino acids and nucleotides standards were acquired from Sigma (St. Louis, MO, USA). Potassium dihydrogen orthophosphate, potassium hydroxide, sodium acetate, di-sodium tetraborate decahydrate, monobasic sodium phosphate, ethylenediaminetetraacetic acid (EDTA), and ascorbic acid were purchased by PanReac (Darmstadt, Germany). All high-performance liquid chromatography (HPLC) reagents, including acetonitrile (ACN), methanol (MeOH), and ethanol (EtOH), were obtained from Scharlau (Barcelona, Spain) in analytical grade. Commercial standards of an α glutamyl dipeptide, α-Glu-Glu; synthetic γ-glutamyl dipeptides and tripeptides, γ-Glu-Glu, γ-Glu-Gln, γ-Glu-Gly, γ-Glu-His, γ-Glu-Val, γ-Glu-Leu, γ-Glu-Met, γ-Glu-Phe, γ-Glu-Tyr, γ-Glu-Val-Gly, and γ-Glu-Cys-Gly; and leucyl dipeptides, Leu-Glu and Leu-Ala, were acquired from Bachem (Weil am Rhein, Germany). Finally, γ-Glu-Ala was purchased from GLPBio (Montclair, NJ, USA).

### 3.2. Proximate Composition and Physicochemical Properties

#### 3.2.1. Sample Preparation

Three raw porcine livers, directly obtained from a porcine slaughterhouse, were weighted and cut into uniform pieces. Liver pieces were vacuum-packaged and subjected to heat treatment in a thermostatic water bath at 90 °C for 10 min to ensure enzymatic inactivation. Then, samples were cooled using an ice bath and homogenized. The homogenized liver paste was vacuum-packaged and stored at −20 °C until further analysis.

#### 3.2.2. Proximate Composition

Protein content was measured by determining the nitrogen content using the Dumas combustion protocol, as described by the Association of Official Agricultural Chemists (AOAC) method 992.15 [46]. The moisture content was quantified with a thermogravimetric balance (HB43 Halogen, Mettler Toledo, Columbus, OH, USA) following the manufacturer’s instructions. All measurements were performed in triplicate. Proximate composition results are shown as g/100 g wet matter with mean values reported.

#### 3.2.3. Physico-Chemical Parameters

Water activity (a_w_) and pH measurements were conducted at 25 °C with an a_w_ meter (Aqualab^®^ 4Tev, Meter Food, Los Angeles, CA, USA) and a meat pH meter (HI 99163, HANNA Instruments, Woonsocket, RI, USA), respectively. Color parameters (luminosity (L*), redness (a*), and yellowness (b*)) were extracted using a Minolta handheld colorimeter (CR-400, Konica Minolta, Tokyo, Japan), calibrated with a white standard calibration plate (L* = 97.43, a* = 4.38, b* = 4.09). Subsequently, hue angle and saturation (Chroma) were calculated:

$$Hue\;\left(h{}^{\circ}\right)=arctan\;(\frac{b*}{a*})$$

$$Chroma\;\left(C*\right)=\sqrt{{a*}^{2}+{b*}^{2}}$$

All measurements were performed in triplicate, and mean values are reported.

### 3.3. Enzymatic Hydrolysis Procedure and Post-Treatments

#### 3.3.1. Sample Preparation

Porcine liver pastes were diluted with double-distilled water to a final concentration of 500 mg/mL and homogenized for 5 min at 4 °C using a masticator homogenizer (IUL Masticator Classic Panoramic, 400 mL, Microplanet, Barcelona, Spain).

#### 3.3.2. Enzymatic Hydrolysis

The hydrolysis procedure was carried out in triplicate following the conditions of a previous work [20]. For the first hydrolysis, 3 mL of 500 mg/mL homogenized solution of porcine liver was hydrolyzed with Alcalase 4.0L Pure at a 1:50 enzyme: substrate ratio for 120 min at 65 °C under constant agitation of 500 rpm. For sequential hydrolyzates, Flavourzyme 1000L or Protana Prime was added at a 1:20 enzyme: substrate ratio and samples were further hydrolyzed for 120 min at 50 °C under constant stirring of 500 rpm. All hydrolyzates were thermally heated at 85 °C for 10 min to achieve enzyme inactivation, cooled in an ice bath, and stored at −20 °C until use.

#### 3.3.3. Transpeptidase Activity

The evaluation of transpeptidase activity was conducted following the Li et al. (2020) method [26], with minor modifications. A 50 mmol/L Na_2_B_4_O_7_-NaOH buffer (pH 10.0) was prepared as the reaction medium. Substrate solutions of 10 mmol/L γ-GpNA and 40 mmol/L Gly-Gly were freshly prepared. Protana Uboost was diluted 1:1600, 1:3200, and 1:6400 (*v*/*v*) prior to use. The reaction mixture contained 500 μL of 10 mmol/L γ-GpNA, 500 μL of 40 mmol/L Gly-Gly (or 50 mmol/L Na_2_B_4_O_7_–NaOH buffer when preparing the negative control), and 20 μL of enzyme solution (diluted Protana Uboost) or undiluted enzyme (Alcalase 4.0L, Flavourzyme 1000L, and Protana Prime). The mixture was incubated at 37 °C for 30 min and subsequently, the reaction ended by adding 480 μL of 0.1 mol/L HCl.

Absorbance was measured at 410 nm using an UV–visible spectrophotometer (Cary 60 UV–visible spectrophotometer, Agilent Technologies, Santa Clara, CA, USA). One unit (U) of enzyme activity was defined as the concentration of enzyme required to release 1 μmol of p-nitroaniline per min from γ-GpNA via the transpeptidation reaction and was determined from the absorbance difference at 410 nm between the sample and the blank.

#### 3.3.4. Substrate Specificity of Protana Uboost

The analysis of substrate specificity of Protana Uboost was conducted following the Li et al. (2022) procedure with modifications [8]. A mixture containing 19 free amino acids (Asp, Glu, Ser, Asn, Gly, Thr, His, Ala, Arg, Pro, Tyr, Met, Val, Ile, Leu, Phe, Trp, Orn, and Lys) was prepared at a final concentration of 10 mmol/L for each amino acid. Glutamine (Gln) was included at a final concentration of 20 mmol/L, since at this concentration Gln can effectively act as both a γ-glutamyl donor and acceptor, according to other authors [26,39]. All amino acids were dissolved in 0.1 mol/L HCl, and the pH of the mixture was adjusted to 9.0 using 2 mol/L NaOH. Reactions were performed in triplicate by adding Protana Uboost (0.2 U/mL or 2 U/mL) to 3 mL of the amino acid mixture. These enzyme concentrations were selected as follows: 0.2 U/mL was chosen to enable direct comparison with the reference study, whereas 2 U/mL was selected to assess whether the effect was enhanced by a tenfold increase in enzyme concentration, as reported by Li et al. (2020) [26]. The samples were incubated at 55 °C for 360 min with constant agitation at 500 rpm. Enzymatic reactions were terminated by heating at 85 °C for 10 min to inactivate the enzyme, followed by rapid cooling in an ice bath. The samples were then stored at −20 °C until further analysis.

#### 3.3.5. Enzymatic γ-Glutamylation Procedure

The enzymatic γ-glutamylation was carried out according to Li et al. (2022 modified protocol) [8]. The pH of the samples was adjusted to 9.0 with NaOH 2 mol/L. Subsequently, the enzymatic synthesis of γ-glutamyl peptides was performed in triplicate by adding Protana Uboost at an enzyme concentration of 0.2 U/mL in 3 mL of sample with or without 20 mmol/L glutamine and incubating at 55 °C for 360 min with constant stirring at 500 rpm. Reactions were terminated by heat treatment at 85 °C for 10 min and then samples were cooled in an ice bath and stored at −20 °C until use.

### 3.4. Degree of Hydrolysis

The degree of hydrolysis (DH) was determined following the OPA method of Nielsen et al. (2001) [47]. Briefly, 18 µL of 5 mg/mL hydrolyzate was mixed with 135 µL of o-phthaldialdehyde (OPA) reagent and incubated at 25 °C for 2 min. Double-distilled water was used as the blank and a serine solution (0.9516 meqv/L) was used as the standard. Absorbance was recorded at 340 nm using a microplate reader (CLARIOstar^®^ Plus, BMG Labtech, Germany).

DH was calculated using the equation

DH (%) = h/h_tot_ × 100

where h_tot_ represents the total number of peptide bonds per protein equivalent, which is 7.60 for meat raw materials, and h denotes the number of hydrolyzed peptide bonds and is calculated as follows:

h = Serine-NH_2_ − β/α

$$Serine-NH2=\frac{\left(Abs340nm\;sample-Abs340nm\;blank\right)}{\left(Abs340nm\;standard-Abs340nm\;blank\right)}*0.9516\;meqv/L*V*100/X*P$$

where serine-NH_2_ = meqv serine NH_2_/g protein; X = g sample; P = protein content in sample expressed in g/100 g; V is the sample volume in L; and α and β are specific values for meat sources (α = 1.00; β = 0.40).

All measurements were performed in triplicate, and mean values are reported.

### 3.5. Determination of Taste-Related Substances

#### 3.5.1. Nucleotides Determination

The method of López-Martínez et al., (2023) [16] was carried out for the extraction of total nucleotides. A total of 30 mL of 0.6 mol/L HClO_4_ was mixed with 8 g of porcine liver paste and homogenized in a stomacher homogenizer (IUL Mod. Masticator Classic Panoramic, 400 mL, Microplanet, Spain) for 4 min at 4 °C. Mixtures were centrifuged at 24,500× *g* for 20 min at 4 °C and subsequently filtered through glass wool at 25 °C. The filtrate was cooled in an ice bath, and the pH was adjusted to 6.5 with 3 mol/L K_2_CO_3_. Finally, samples were centrifuged again (24,500× *g*, 10 min, 4 °C), mixed with acetonitrile (1:1, *v*/*v*), and filtered through a 0.22 μm nylon membrane.

Total nucleotide analysis was conducted following the method of Mora et al. (2010) [48] using hydrophilic interaction chromatography (HILIC) with a ZIC^®^-pHILIC column (4.6 × 150 mm, 5 μm) coupled with a guard column (2.1 × 20 mm, 5 μm) on an HPLC system (Acquity Arc CH/CHC Core Fluidics; Waters, Milford, MA, USA). Chromatographic conditions were set as follows: column temperature, 28 °C; flow rate, 0.5 mL/min; and detection wavelength, 254 nm. Mobile phases consisted of (A) bidistilled water, (B) acetonitrile, and two ammonium acetate buffers ((C) 150 mmol/L, pH 3.5; and (D) 100 mmol/L, pH 7). Nucleotides were quantified using standard calibration curves, and the results are expressed as mg/100 g wet matter. All determinations were performed in triplicate, and mean values are reported.

#### 3.5.2. Free Amino Acids (FAAs) Determination

In total, 250 μL of 5 mg/mL dilution of samples was mixed with 40 μL of the internal standard (5 mmol/L norleucine) and 750 μL of ACN. Mixtures were derivatized following the Pico-tag protocol of Aristoy & Toldra, (1991) [49]. Chromatographic analysis was conducted according to Flores et al. (1997) [50] on an HPLC system (Acquity Arc CH/CHC Core Fluidics; Waters, USA) with a reverse-phase Waters Pico Tag^®^ C18 column (60 Å, 4 µm, 3.9 mm × 300 mm; Waters Corp., USA) using two mobile phases, ACN:H_2_O:MeOH (45:40:15) and 70 mmol/L sodium acetate buffer with 2.5% ACN (*v*:*v*) (pH 6.55), with the following conditions: column temperature of 52 °C, sample temperature of 12 °C, flow rate of 1 mL/min, and detection wavelength at 254 nm. Free amino acids were quantified using standard curves, and results are expressed as mg FAA/g wet matter. All assays were performed in triplicate, and mean values are reported. The summatory of Threonine (Thr), Lysine (Lys), Isoleucine (Ile), Leucine (Leu), Valine (Val), Tryptophan (Trp), Histidine (His) and Methionine (Met) was expressed as the total essential amino acids content (EAAs), while the summatory of Ile, Leu and Val corresponds to the branched-chain amino acid content (BCAAs). Taste attributes of amino acids (umami: Asp and Glu; sweet: Ser, Gly, Gln, Thr and Ala; bittersweet: Arg, Pro, Val, Met, Cys, Lys; and bitter: Tau, His, Tyr, Ile, Leu, Phe and Trp), were compiled using data from the previous literature [17,51,52,53].

#### 3.5.3. Equivalent Umami Concentration

The potential synergistic effect of the umami-enhancing perception of free amino acids and nucleotides was evaluated with the equivalent umami concentration (EUC) [18] and calculated with the following equation

EUC (g MSG/100 g organ) =Ʃa_i_b_i_ + 1218 Ʃa_i_b_i_ (Ʃa_j_b_j_)

where a_i_ and a_j_ denote the concentrations of umami-related free amino acids (Asp and Glu) and umami-related nucleotides (IMP, GMP, AMP), respectively, while b_i_ and b_j_ represents their relative umami contributions (RUC) in comparison to monosodium glutamate (MSG) (Asp = 0.077, Glu = 1) for free amino acids and to inosine monophosphate (IMP) (IMP = 1, GMP = 2.3, AMP = 0.18) for nucleotides. The constant 1218 represents the synergistic enhancement of umami intensity resulting from the interaction between umami amino acids and nucleotides. Results are expressed as g MSG/100 g wet matter and mean values are reported.

#### 3.5.4. Taste Activity Value

The quantification of the relative potential influence of each single taste-related compound on the overall taste was conducted by calculating the taste activity value (TAV) with the following formula [17]

$$Taste\;activity\;value\;\left(TAV\right)=\frac{C}{T}$$

where C represents the concentration of each taste-related substance in the sample and T denotes its corresponding taste threshold. A TAV greater than 1 indicates that the compound contributes perceptibly to taste, with higher values reflecting a stronger sensory impact. TAV values are dimensionless, and mean results are reported. Taste threshold values for free amino acids and nucleotides were obtained from previously published sources [17,51,52].

#### 3.5.5. Di- and Tripeptides Quantification

The quantification of di- and tripeptides was performed following the procedure of Mesonzhnik et al. (2020) [54] with some modifications. For derivatization, 100 μL of porcine liver hydrolyzates (20 mg/mL) was mixed with 100 μL of TCEP solution (20 mmol/L TCEP prepared in 131 mmol/L Na_2_B_4_O_7_ buffer, pH 8.8) and incubated for 60 min at 25 °C to reduce disulfide bonds. In total, 10 µL of the reduced sample was mixed with 60 μL Na_2_B_4_O_7_ buffer (131 mmol/L, pH 8.8), 10 μL of internal standard (norvaline 200 µmol/L) and 20 µL of AQC derivatization solution (15 mmol/L AQC in acetonitrile). The mixtures were vortexed immediately, left for 1 min, and heated at 55 °C for 10 min under constant agitation of 500 rpm. Subsequently, samples were cooled to 25 °C and centrifuged at 14,000× *g* for 10 min at 4 °C.

For γ Glu-Cys-Gly quantification, the standard derivatization procedure was not applicable due to reagent interference. Instead, 20 mg/mL hydrolyzates were diluted with 10 mmol/L of DTT and incubated for 60 min at 25 °C, followed by centrifugation at 14,000× *g* for 10 min at 4 °C.

RP-UHPLC-PDA-MS analysis was performed using a Thermo Vanquish UHPLC system (Thermo Scientific, San Jose, CA, USA) coupled to an Orbitrap IQ-X Tribrid mass spectrometer (Thermo Scientific, San Jose, CA). Separation was achieved on a Waters Acquity HSS T3 column (2.1 × 150 mm i.d., 1.8 μm particle size) using HPLC-grade water (A) and acetonitrile (B), both acidified with 1% (*v*:*v*) formic acid (0.1% (*v*:*v*) for γ Glu-Cys-Gly assays). The flow rate was 400 μL/min, and the column temperature was 45 °C. A total of 2 μL of sample (4 μL in the case of γ Glu-Cys-Gly) was injected. The elution gradient was as follows: 0–2.0 min isocratic at 2% B, 2.0–25.0 min linear gradient from 2 to 35% B, 25.0–30.0 min linear gradient from 35 to 95% B, 30.0–33.0 min isocratic at 95% B, 33.0–33.1 min linear gradient to starting conditions, and 33.1–40.0 min isocratic at 2% B. PDA detection was performed at 260 nm (20 Hz). Nitrogen was used as both sheath gas (50 arbitrary units) and auxiliary gas (10 arbitrary units). The ion source was operated at a capillary temperature of 325 °C, a spray voltage of 3.5 kV, and an S-Lens RF level of 50%. Accurate mass acquisition was performed at an *m*/*z* range of 190–800. Full MS measurements scans were acquired at 30,000 FWHM. Confirmation was performed by HCD resolution combined with fragmentation by HCD with an NCE between 15 and 45, for which MS2 data were recorded at 30,000 FWHM resolution. Thereby, the software increased the collision energy starting from NCE 15 stepwise with stepping energies (20, 25, 30, 35, 40, and 45) using the MSn qQuality trigger of unfragmented precursors below 20%. Precursor ions were, for this measurement, a mass list containing the masses of the leucyl dipeptides, glutamyl-dipeptides and tripeptides of interest derivatized with AQC. All precursor ions were sourced from the University of Washington’s Proteomics Resource (UWPR) using the peptide fragmentation tool (https://proteomicsresource.washington.edu/cgi-bin/fragment.cgi) (accessed on 18 October 2025). The quantitation used full MS scans at 30,000 FWHM. Data processing was performed using Xcalibur 4.5 and Freestyle 1.8 (Thermo Scientific). Di- and tripeptides were quantified using external calibration curves (Table S1), and results are expressed as μg peptide/g wet matter. Koku-related peptide content was considered the sum of γ-glutamyl peptides and leucyl peptides. All assays were performed in triplicate, and mean values are reported.

### 3.6. RP-HPLC Peptide Profiles

A total of 300 μL of 500 mg/mL dilutions was deproteinized according to Mora et al. (2015) with 900 μL of EtOH, incubated for 960 min at 4 °C and followed by a centrifugation at 20,879× *g* for 5 min at 4 °C [55]. Then, 900 μL of each supernatant was vacuum-dried (SpeedVac SPD120, Thermo Fisher, Waltham, MA, USA) at 35 °C for 300 min. Dried samples were rehydrated with 250 μL of double-distilled water and filtered through a 0.45 μm nylon membrane.

Peptide profile separation by hydrophobicity was assessed following the Gallego et al. (2024) method [56] using reversed-phase HPLC (Agilent 1100, Agilent Technologies, USA) with a Symmetry C18 column (4.6 × 250 mm, 5 μm; Waters, USA) at 25 °C. Solvent A was 0.1% TFA in double-distilled water, and solvent B was 0.085% TFA in acetonitrile: double-distilled water (60:40, *v*/*v*). Both solvents were filtered through a 0.45 μm membrane and degassed. In total, 10 μL of sample was eluted at a 1 mL/min flow rate with a gradient from 0 to 50% B during 50 min and a detection wavelength of 214 nm.

### 3.7. Biological Activity Assays

#### 3.7.1. Sample Preparation

In total, 0.4 mg/mL unhydrolyzed samples or 0.1 mg/mL hydrolyzates were used for an Oxygen Radical Antioxidant Capacity (ORAC) assay. For DPPH, ferric reducing antioxidant power (FRAP) and Fe^2+^ ion chelating power, 100 μL of 500 mg/mL sample solution was deproteinized according to Mora et al. (2015) with 600 μL of EtOH and incubated at 4 °C for 16 h [55]. Subsequently, centrifugation at 20,879× *g* for 5 min at 4 °C was performed and supernatants were stored in Eppendorf tubes at −20 °C until use. For ABTS assays, 1:4 dilutions (*v*:*v*) of deproteinized samples with EtOH were used.

#### 3.7.2. Antioxidant Activity Assays

Five different methods were carried out to evaluate in vitro antioxidant activity: (i) an ABTS assay was conducted based on the Re et al. (1999) method [57], with the results expressed as mmol of Trolox equivalent antioxidant capacity (TEAC) per mg of sample; (ii) a DPPH radical-scavenging assay was performed according to Bersuder et al.’s procedure (1998) [58], presenting the results as the percentage of inhibition; (iii) a FRAP assay was carried out following the methodology of Chen et al. (2010) [59] and the results are expressed as 700 nm Absorbance Units (AU); (iv) an ORAC assay was conducted according to Davalos et al. (2004) [60], reporting the results as mmol of Trolox equivalent per gram of wet matter; (v) an Fe^2+^ ion chelating power assay was performed as described Zheng et al. (2019) [61] with the results as the percentage of chelation. All assays were conducted in triplicate with mean values reported.

### 3.8. Statistical Analysis

Statistical analysis was carried out using one-way analysis of variance (ANOVA), followed by Tukey’s rank test, with a significance threshold of *p* < 0.05. Pearson correlation analyses were carried out on biological activities and degree of hydrolysis. Minitab Statistical Software v22.4 (Minitab, LLC, State College, PA, USA) was the statistical software used for these analyses.

## 4. Conclusions

In conclusion, post-treatment with Protana Uboost at 0.2 U/mL in porcine liver hydrolyzates has been shown to be an effective strategy for its valorization, generating γ-glutamyl dipeptides with koku potential. The results of this study suggest that sequential hydrolysis with Alcalase and Protana Prime, followed by post-treatment with Protana Uboost, is a promising combination for generating koku peptides and obtaining the highest antioxidant capacity values, achieving an adequate balance for the formation of hydrolyzates that can be considered as both potential taste-enhancers and functional ingredients at the same time. Future analysis including quantitation of additional γ-glutamyl peptide sequences would permit us to confirm the influence of Gln addition. Finally, sensory analysis would support the mechanism of action and taste-enhancing capacity of these peptides. The approach taken in this study demonstrated a promising route to valorize co-products of the porcine meat industry, like livers, into tasty and functional food ingredients, which can be used for the development of food products with reduced salt and sugar content, making them healthier without compromising palatability.

## Figures and Tables

**Table 1:** Codification of the hydrolyzates produced under the different conditions tested.

HC ^a^	Dilution 500 mg/mL of porcine liver with double-distilled water
HA	Porcine liver hydrolyzate with Alcalase 4P 1/50 E/S ratio
HAF	Porcine liver hydrolyzate with Alcalase 4P 1/50 E/S ratio + Flavourzyme 1000L 1/20 E/S ratio
HAPP	Porcine liver hydrolyzate with Alcalase 4P 1/50 E/S ratio + Protana Prime 1/20 E/S ratio
HCU	Dilution 500 mg/mL of porcine liver with double-distilled water + Protana Uboost 0.2 U/mL
HAU	Porcine liver hydrolyzate with Alcalase 4P 1/50 E/S ratio + Protana Uboost 0.2 U/mL
HAFU	Porcine liver hydrolyzate with Alcalase 4P 1/50 E/S ratio + Flavourzyme 1000L 1/20 E/S ratio + Protana Uboost 0.2 U/mL
HAPPU	Porcine liver hydrolyzate with Alcalase 4P 1/50 E/S ratio + Protana Prime 1/20 E/S ratio + Protana Uboost 0.2 U/mL
HCUG	Dilution 500mg/mL of porcine liver with double-distilled water + Protana Uboost 0.2 U/mL + Gln 20 mmol/L
HAUG	Porcine liver hydrolyzate with Alcalase 4P 1/50 E/S ratio + Protana Uboost 0.2 U/mL + Gln 20 mmol/L
HAFUG	Porcine liver hydrolyzate with Alcalase 4P 1/50 E/S ratio + Flavourzyme 1000L 1/20 E/S ratio + Protana Uboost 0.2 U/mL+ Gln 20 mmol/L
HAPPUG	Porcine liver hydrolyzate with Alcalase 4P 1/50 E/S ratio + Protana Prime 1/20 E/S ratio + Protana Uboost 0.2 U/mL + Gln 20 mmol/L
STD0	Free amino acids solution 10 mmol/L pH 4
STDH	Free amino acids solution 10 mmol/L pH 9 6 h 55 °C
STDU0.2	Free amino acids solution 10 mmol/L pH 9+ Protana Uboost 0.2 U/mL 6 h 55 °C
STDU2	Free amino acids solution 10 mmol/L pH 9+ Protana Uboost 2 U/mL 6 h 55 °C

^a^ C, control or unhydrolyzed sample; A, Alcalase 4.0L Pure hydrolyzate; F, Flavourzyme1000 L hydrolyzate; PP, Protana Prime hydrolyzate; U, Protana Uboost posttreatment; G, addition of free glutamine at a final concentration of 20 mmol/L; 0, no treatment; H, heat treatment; 0.2 and 2, Protana Uboost at a final enzyme activity of 0.2 U/mL and 2 U/mL, respectively.

**Table 2:** Degree of hydrolysis (DH) in percentage, free amino acids content (mg amino acids/g wet matter) and equivalent umami content (EUC) expressed as g monosodium glutamate (MSG)/100 g wet matter of porcine liver hydrolyzates (N = 3, mean ± SEM).

	HC ^a^	HA	HAF	HAPP	HCU	HAU	HAFU	HAPPU	HCUG	HAUG	HAFUG	HAPPUG
	Mean ± SEM	Mean ± SEM	Mean ± SEM	Mean ± SEM	Mean ± SEM	Mean ± SEM	Mean ± SEM	Mean ± SEM	Mean ± SEM	Mean ± SEM	Mean ± SEM	Mean ± SEM
DH (%)	9.33 ± 2.33 eB	43.78 ± 0.65 cB	54.72 ± 0.95 bA	63.92 ± 1.73 aA	16.45 ± 1.82 dA	46.15 ± 1.78 cAB	56.61 ± 0.11 bA	60.07 ± 1.58 abA	16.52 ± 0.70 dA	48.83 ± 1.01 cA	55.83 ± 1.02 bA	64.65 ± 4.81 aA
Asp	0.52 ± 0.03 fgB ^b^	0.76 ± 0.11 defA	1.39 ± 0.11 cA	3.11 ± 0.24 aA	0.74 ± 0.03 efA	0.81 ± 0.02 defA	1.07 ± 0.15 dB	2.42 ± 0.09 bB	0.42 ± 0.03 gC	0.64 ± 0.11 fgA	1.04 ± 0.09 deB	2.45 ± 0.03 bB
Glu	2.16 ± 0.07 gB	3.90 ± 0.26 defAB	5.12 ± 0.41 bcA	7.31 ± 0.46 aA	3.62 ± 0.29 efA	4.37 ± 0.33 cdeA	5.48 ± 0.24 bA	6.77 ± 0.36 aA	3.09 ± 0.35 fgA	3.29 ± 0.26 fB	4.68 ± 0.32 bcdA	6.64 ± 0.54 aA
**Umami aas**	**2.68 ± 0.09 gC**	**4.66 ± 0.28 defAB**	**6.51 ± 0.52 cA**	**10.43 ± 0.69 aA**	**4.36 ± 0.31 defA**	**5.18 ± 0.33 deA**	**6.55 ± 0.18 cA**	**9.19 ± 0.32 bA**	**3.52 ± 0.37 fgB**	**3.93 ± 0.28 fB**	**5.72 ± 0.39 cdA**	**9.09 ± 0.55 bA**
Ser	0.45 ± 0.02 fB	2.34 ± 0.31 deA	4.12 ± 0.50 cA	6.05 ± 0.59 aA	0.60 ± 0.04 fA	2.43 ± 0.19 deA	3.13 ± 0.14 dB	4.26 ± 0.09 bB	0.39 ± 0.02 fB	2.08 ± 0.18 eA	3.06 ± 0.14 dB	4.96 ± 0.33 bB
Gly	1.17 ± 0.01 fgB	1.58 ± 0.11 defgAB	2.39 ± 0.20 cA	4.14 ± 0.13 aA	1.60 ± 0.19 defA	1.44 ± 0.11 defgAB	1.82 ± 0.13 deB	2.84 ± 0.14 bcB	1.10 ± 0.09 gB	1.33 ± 0.05 efgB	1.84 ± 0.20 dB	3.18 ± 0.36 bB
Gln	0.51 ± 0.05 fB	3.89 ± 1.51 dA	4.39 ± 0.52 bcdB	5.89 ± 0.50 abcA	0.56 ± 0.14 fB	0.80 ± 0.16 efB	1.26 ± 0.10 efC	1.62 ± 0.18 efC	2.73 ± 0.16 deA	4.12 ± 1.41 cdA	6.20 ± 0.23 abA	7.28 ± 0.48 aA
Thr	0.60 ± 0.09 gA	3.72 ± 0.69 defA	6.47 ± 0.58 bA	8.40 ± 0.75 aA	0.53 ± 0.06 gA	2.97 ± 0.12 efA	4.59 ± 0.44 cdB	5.05 ± 0.21 cB	0.51 ± 0.05 gA	2.64 ± 0.33 fA	3.97 ± 0.31 cdB	5.05 ± 0.26 cB
Ala	0.97 ± 0.07 gB	4.43 ± 0.47 fA	6.50 ± 0.58 cdA	9.00 ± 0.56 aA	1.47 ± 0.21 gA	4.66 ± 0.32 efA	6.06 ± 0.06 dA	7.56 ± 0.26 bcB	1.02 ± 0.09 gB	3.84 ± 0.22 fA	5.55 ± 0.44 deA	8.05 ± 0.52 bAB
**Sweet aas**	**3.70 ± 0.11 gC**	**15.97 ± 1.31 efA**	**23.87 ± 2.10 cA**	**33.48 ± 2.41 a**	**4.76 ± 0.54 gB**	**12.31 ± 0.65 fB**	**16.85 ± 0.60 deB**	**21.32 ± 0.72 c**	**5.75 ± 0.37 gA**	**14.01 ± 1.20 efAB**	**20.63 ± 1.23 cdA**	**28.52 ± 1.86 b**
Arg	0.14 ± 0.03 cA	0.91 ± 0.09 bA	2.87 ± 0.30 aA	3.09 ± 0.44 aA	0.21 ± 0.13 cA	0.94 ± 0.05 bA	3.20 ± 0.33 aA	2.67 ± 0.11 aA	0.11 ± 0.01 cA	0.81 ± 0.08 bA	2.86 ± 0.21 aA	2.66 ± 0.12 aA
Pro	0.35 ± 0.02 bcA	0.28 ± 0.07 bcdeA	0.37 ± 0.07 bA	0.52 ± 0.05 aA	0.34 ± 0.05 bcA	0.23 ± 0.01 cdeA	0.25 ± 0.02 bcdeB	0.35 ± 0.02 bcB	0.31 ± 0.04 bcdeA	0.21 ± 0.06 deA	0.20 ± 0.02 eB	0.33 ± 0.02 bcdB
Val	0.46 ± 0.05 fA	5.16 ± 0.66 deA	8.39 ± 0.93 bA	10.50 ± 0.91 aA	0.45 ± 0.07 fA	4.80 ± 0.29 eA	7.18 ± 0.32 bcAB	7.59 ± 0.27 bcB	0.42 ± 0.03 fA	4.10 ± 0.48 eA	6.30 ± 0.39 cdB	7.62 ± 0.40 bcB
Met	0.21 ± 0.03 gA	2.70 ± 0.21 defA	3.84 ± 0.49 bA	4.63 ± 0.42 aA	0.19 ± 0.03 gA	2.56 ± 0.14 efAB	3.33 ± 0.15 bcdA	3.38 ± 0.11 bcB	0.21 ± 0.03 gA	2.16 ± 0.21 fBA	3.07 ± 0.20 cdeB	3.52 ± 0.19 bcB
Cys	0.01 ± 0.00 dA	0.80 ± 0.26 bcA	0.85 ± 0.18 bcA	1.73 ± 0.28 aA	0.01 ± 0.00 dA	0.72 ± 0.12 bcA	0.95 ± 0.03 bcA	0.95 ± 0.17 bB	0.02 ± 0.00 dA	0.58 ± 0.02 cA	0.75 ± 0.12 bcA	1.02 ± 0.07 bcB
Lys	0.65 ± 0.04 fA	4.17 ± 0.50 eA	8.60 ± 0.69 bA	11.21 ± 0.72 aA	0.77 ± 0.25 fA	3.90 ± 0.36 eA	7.35 ± 0.23 cdAB	8.02 ± 0.28 bcB	0.56 ± 0.03 fA	3.44 ± 0.26 eA	6.79 ± 0.48 dB	8.33 ± 0.44 bcB
**Bittersweet aas**	**1.81 ± 0.09 eA**	**14.02 ± 1.72 dA**	**24.92 ± 2.51 bA**	**31.69 ± 2.59 aA**	**1.97 ± 0.44 eA**	**13.15 ± 0.91 dA**	**22.27 ± 1.02 bcAB**	**22.96 ± 0.89 bcB**	**1.62 ± 0.11 eA**	**11.31 ± 0.92 dA**	**19.97 ± 1.29 cB**	**23.47 ± 1.16 bcB**
Tau	1.00 ± 0.04 aA	0.76 ± 0.03 abAB	0.79 ± 0.11 abA	1.00 ± 0.10 aA	1.05 ± 0.29 aA	0.80 ± 0.02 abA	0.81 ± 0.03 abA	0.78 ± 0.05 abA	0.75 ± 0.03 abA	0.69 ± 0.05 bB	0.82 ± 0.03 abA	0.85 ± 0.12 abA
His	0.22 ± 0.03 dA	1.86 ± 0.08 cA	3.11 ± 0.20 bA	4.13 ± 0.48 aA	0.19 ± 0.10 dA	1.83 ± 0.16 cA	2.82 ± 0.14 bAB	2.96 ± 0.20 bB	0.22 ± 0.03 dA	1.57 ± 0.13 cA	2.57 ± 0.16 bB	3.13 ± 0.20 bB
Tyr	0.30 ± 0.05 fA	4.09 ± 0.32 deA	5.93 ± 0.62 bA	7.30 ± 0.74 aA	0.22 ± 0.03 fA	3.97 ± 0.28 deAB	5.25 ± 0.16 bcAB	5.54 ± 0.30 bcB	0.21 ± 0.06 fA	3.34 ± 0.12 eB	4.76 ± 0.26 cdB	5.64 ± 0.24 bcB
Ile	0.37 ± 0.02 gA	3.54 ± 0.78 defA	6.22 ± 0.72 bA	7.62 ± 0.70 aA	0.37 ± 0.07 gA	3.07 ± 0.28 efA	4.96 ± 0.13 cB	4.71 ± 0.35 cdB	0.30 ± 0.02 gA	2.61 ± 0.46 fA	4.12 ± 0.22 cdB	4.68 ± 0.23 cdB
Leu	0.75 ± 0.06 dA	9.77 ± 0.39 cA	14.60 ± 1.41 bA	16.93 ± 1.33 aA	0.95 ± 0.19 dA	9.59 ± 0.71 cAB	13.79 ± 0.66 bA	13.38 ± 0.77 bB	0.80 ± 0.07 dA	8.35 ± 0.37 cB	12.69 ± 0.90 bA	14.33 ± 0.84 bB
Phe	0.40 ± 0.05 eAB	5.08 ± 0.89 bcdA	7.96 ± 0.54 aA	9.32 ± 0.79 aA	0.47 ± 0.06 eA	4.54 ± 0.35 cdA	6.32 ± 0.50 bB	6.02 ± 0.36 bB	0.34 ± 0.03 eB	3.96 ± 0.59 dA	5.48 ± 0.40 bB	6.13 ± 0.41 bB
Trp	0.25 ± 0.04 eA	1.83 ± 0.42 dA	2.70 ± 0.17 abA	3.34 ± 0.49 aA	0.31 ± 0.06 eA	1.97 ± 0.21 cdA	2.75 ± 0.20 abA	2.74 ± 0.16 abA	0.28 ± 0.06 eA	1.63 ± 0.19 dA	2.52 ± 0.17 bA	3.00 ± 0.05 abA
**Bitter aas**	**3.29 ± 0.10 fA**	**26.93 ± 2.06 eA**	**41.33 ± 3.73 bA**	**49.65 ± 4.44 aA**	**3.55 ± 0.63 fA**	**25.76 ± 1.97 eAB**	**36.70 ± 1.37 bcAB**	**36.14 ± 1.93 bcB**	**2.91 ± 0.23 fA**	**22.14 ± 1.43 eB**	**32.95 ± 2.09 cdB**	**37.75 ± 1.98 bc**
Hyp	0.02 ± 0.00 cA	0.06 ± 0.01 abcA	0.08 ± 0.03 aA	0.08 ± 0.01 aA	0.02 ± 0.02 bcA	0.07 ± 0.01 aA	0.08 ± 0.01 aA	0.06 ± 0.01 abA	0.02 ± 0.00 bcA	0.05 ± 0.01 abcA	0.07 ± 0.01 aA	0.08 ± 0.03 aA
Asn	0.57 ± 0.08 gA	3.99 ± 0.51 efA	6.60 ± 0.63 bcA	8.88 ± 0.41 aA	0.66 ± 0.25 gA	3.44 ± 0.11 fAB	5.70 ± 0.22 cdAB	6.91 ± 0.46 bB	0.46 ± 0.09 gA	3.12 ± 0.28 fB	4.95 ± 0.37 deB	6.58 ± 0.15 bcB
β-Ala	0.09 ± 0.01 abA	0.06 ± 0.02 bA	0.05 ± 0.00 bC	0.06 ± 0.01 bA	0.09 ± 0.01 abA	0.09 ± 0.02 abA	0.12 ± 0.02 abB	0.08 ± 0.04 abA	0.10 ± 0.01 abA	0.10 ± 0.01 abA	0.16 ± 0.02 aA	0.13 ± 0.06 abA
Orn	0.40 ± 0.01 eB	1.88 ± 0.13 dA	3.13 ± 0.25 bA	3.70 ± 0.36 aA	0.55 ± 0.01 eA	1.76 ± 0.17 dA	2.73 ± 0.11 bcAB	2.85 ± 0.09 bcB	0.39 ± 0.02 eB	1.58 ± 0.12 dA	2.50 ± 0.17 cB	2.93 ± 0.10 bcB
HLys	0.05 ± 0.02 bcB	0.48 ± 0.06 abcA	0.70 ± 0.24 aA	0.79 ± 0.45 aA	0.02 ± 0.01 cB	0.31 ± 0.07 abcA	0.41 ± 0.15 abcA	0.34 ± 0.07 bcA	0.17 ± 0.04 bcA	0.51 ± 0.14 abcA	0.48 ± 0.24 abcA	0.56 ± 0.06 abA
**Taste aas**	**11.48 ± 0.29 fB**	**61.58 ± 4.69 eA**	**96.63 ± 8.81 bcA**	**125.25 ± 10.09 aA**	**14.65 ± 1.83 fA**	**56.40 ± 3.86 eAB**	**82.37 ± 3.04 cdAB**	**89.61 ± 3.79 bcdB**	**13.80 ± 1.07 fAB**	**51.39 ± 2.80 eB**	**79.27 ± 4.93 dB**	**98.84 ± 5.53 bB**
**EAAs**	**3.45 ± 0.11 eA**	**32.66 ± 3.09 dA**	**53.52 ± 4.56 bA**	**65.59 ± 5.56 aA**	**3.77 ± 0.68 eA**	**30.42 ± 2.30 dA**	**45.91 ± 1.99 bcAB**	**46.28 ± 2.21 bcB**	**3.22 ± 0.27 eA**	**26.36 ± 2.04 dA**	**41.21 ± 2.64 cB**	**48.17 ± 2.55 bcB**
**BCAAs**	**1.58 ± 0.13 fA**	**18.48 ± 1.81 deA**	**29.21 ± 3.06 bA**	**35.06 ± 2.89 aA**	**1.76 ± 0.31 fA**	**17.46 ± 1.27 eA**	**25.93 ± 1.08 bcAB**	**25.68 ± 0.33 bcB**	**1.52 ± 0.10 fA**	**15.06 ± 1.18 eA**	**23.10 ± 1.46 cdB**	**26.62 ± 1.46 bcB**
**Total aas**	**12.63 ± 0.31 fA**	**68.12 ± 5.32 eA**	**107.25 ± 9.86 bcA**	**138.85 ± 11.01 aA**	**16.00 ± 2.04 fA**	**62.18 ± 4.18 eAB**	**91.53 ± 3.10 bcdAB**	**99.98 ± 4.39 bcB**	**15.00 ± 1.18 fA**	**56.84 ± 3.06 eB**	**87.58 ± 5.48 dB**	**109.27 ± 5.70 bB**
**EUC**	**32.94 ± 4.11 eB**	**59.55 ± 10.75 cdeA**	**78.33 ± 10.86 bcA**	**113.89 ± 22.35 aA**	**55.42 ± 11.00 cdeA**	**66.31 ± 8.68 cdeA**	**83.35 ± 10.83 bA**	**103.91 ± 9.23 abA**	**46.97 ± 8.47 deAB**	**49.78 ± 4.37 cdeA**	**71.55 ± 12.36 bcdA**	**103.60 ± 20.96 abA**

^a^ C, control or unhydrolyzed sample; A, Alcalase 4.0L Pure hydrolyzate; F, Flavourzyme1000L hydrolyzate; PP, Protana Prime hydrolyzate; U, Protana Uboost post-treatment; G, addition of free glutamine at final concentration of 20 mmol/L; 0, no treatment. ^b^ Lowercase different letters (a, b, c, d, e, f, g) represents significant differences (*p* < 0.05) among all samples, while uppercase different letters (A, B, and C) reflect significant differences (*p* < 0.05) within the same group (unhydrolyzed samples: HC, HCU, and HCUG; single Alcalase hydrolyzates: HA, HAU, and HAUG; sequential Alcalase Flavourzyme hydrolyzates: HAF, HAFU, and HAFUG; and sequential Alcalase Protana Prime hydrolyzates: HAPP, HAPPU, and HAPPUG).

**Table 3:** Taste active values (TAV, dimensionless) of free amino acids of porcine liver hydrolyzates expressed by means.

Amino Acid	Taste Threshold (mg aas/g Wet Matter)	Taste Attribute	HC ^b^	HA	HAF	HAPP	HCU	HAU	HAFU	HAPPU	HCUG	HAUG	HAFUG	HAPPUG
Asp	1.0	Umami	0.52	0.76	1.39 ^a^	3.11	0.74	0.82	1.07	2.42	0.42	0.64	1.04	2.45
Glu	0.3	Umami	7.19	13.00	17.07	24.38	12.07	14.56	18.27	22.57	10.32	10.95	15.60	22.16
Ser	1.5	Sweet	0.30	1.56	2.75	4.04	0.40	1.62	2.08	2.84	0.26	1.39	2.04	3.31
Gly	1.3	Sweet	0.90	1.22	1.84	3.19	1.23	1.11	1.40	2.18	0.85	1.02	1.41	2.45
Thr	2.6	Sweet	0.05	0.04	0.04	0.05	0.06	0.04	0.04	0.04	0.04	0.04	0.04	0.05
Ala	0.6	Sweet	1.08	9.28	15.57	20.67	0.94	9.13	14.11	14.81	1.09	7.87	12.87	15.66
Arg	0.5	Bittersweet	0.23	1.43	2.49	3.23	0.20	1.14	1.77	1.94	0.20	1.02	1.53	1.94
Pro	3	Bittersweet	1.61	7.39	10.83	15.00	2.45	7.77	10.10	12.60	1.71	6.40	9.26	13.41
Val	0.4	Bittersweet	0.27	1.81	5.74	6.19	0.42	1.89	6.41	5.34	0.21	1.63	5.72	5.32
Met	0.3	Bittersweet	0.12	0.09	0.12	0.17	0.11	0.08	0.08	0.12	0.10	0.07	0.07	0.11
Lys	0.5	Bittersweet	0.31	4.24	6.14	7.55	0.23	4.11	5.44	5.74	0.22	3.46	4.93	5.84
Tau	18.8	Bitter	1.15	12.91	20.97	26.25	1.12	12.00	17.96	18.97	1.05	10.26	15.76	19.04
His	0.2	Bitter	0.70	9.00	12.81	15.42	0.62	8.52	11.09	11.28	0.69	7.20	10.23	11.74
Tyr	0.9	Bitter	0.41	3.93	6.92	8.47	0.41	3.41	5.51	5.23	0.34	2.89	4.57	5.20
Ile	0.9	Bitter	0.40	5.14	7.69	8.91	0.50	5.05	7.26	7.04	0.42	4.39	6.68	7.54
Leu	1.9	Bitter	0.45	5.64	8.85	10.36	0.53	5.04	7.02	6.69	0.38	4.40	6.09	6.81
Phe	0.9	Bitter	0.28	2.04	3.00	3.71	0.35	2.19	3.06	3.05	0.31	1.81	2.80	3.33
Trp	0.9	Bitter	1.29	8.34	17.19	22.42	1.54	7.80	14.70	16.03	1.12	6.88	13.58	16.66

^a^ TAV > 1 = 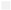; TAV > 5 = 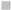; TAV > 10 = 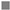; TAV > 15 = 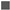; TAV > 20 = 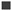. ^b^ C, control or unhydrolyzed sample; A, Alcalase 4.0L Pure hydrolyzate; F, Flavourzyme1000L hydrolyzate; PP, Protana Prime hydrolyzate; U, Protana Uboost post-treatment; G, addition of free glutamine at final concentration of 20 mmol/L; 0, no treatment.

**Table 4:** Quantitated di- and tripeptides (μg peptides/g wet matter) with koku-related potential obtained from porcine liver hydrolyzates (N = 3, mean ± SEM).

	HC ^a^	HA	HAF	HAPP	HCU	HAU	HAFU	HAPPU	HCUG	HAUG	HAFUG	HAPPUG
Peptides	Mean ± SEM	Mean ± SEM	Mean ± SEM	Mean ± SEM	Mean ± SEM	Mean ± SEM	Mean ± SEM	Mean ± SEM	Mean ± SEM	Mean ± SEM	Mean ± SEM	Mean ± SEM
Leu-Glu	0.00 ± 0.00 eA ^b^	18.31 ± 0.42 dB	61.87 ± 4.28 aA	23.12 ± 1.13 dB	0.00 ± 0.00 eA	23.08 ± 1.03 dA	56.26 ± 2.46 bA	33.98 ± 0.57 cA	0.00 ± 0.00 eA	22.72 ± 0.76 dA	55.67 ± 2.99 bA	32.16 ± 1.94 cA
Leu-Ala	0.00 ± 0.00 eB	125.97 ± 3.84 bB	107.89 ± 4.11 cB	67.98 ± 0.22 dC	0.89 ± 0.10 eA	158.29 ± 1.90 aA	119.45 ± 7.01 bA	100.55 ± 2.05 cB	0.95 ± 0.10 eA	152.70 ± 6.70 aA	123.67 ± 1.82 bA	105.68 ± 2.76 cA
**Leucyl peptides**	**0.00 ± 0.00 dB**	**144.76 ± 4.24 bB**	**169.76 ± 8.14 aA**	**91.10 ± 1.26 cB**	**0.89 ± 0.10 dA**	**181.36 ± 2.52 aA**	**175.71 ± 9.45 aA**	**134.53 ± 2.36 bA**	**0.95 ± 0.10 dA**	**175.42 ± 6.96 aA**	**179.64 ± 4.67 aA**	**137.85 ± 4.66 bA**
γ Glu-Glu	14.52 ± 3.71 eB	10.14 ± 0.88 eC	10.61 ± 0.25 eC	8.43 ± 0.28 eC	9.93 ± 0.28 eB	94.96 ± 1.64 dB	121.60 ± 6.61 cdA	197.29 ± 5.80 bA	513.93 ± 34.10 aA	137.85 ± 16.23 cC	92.75 ± 1.07 dB	109.29 ± 3.90 cdB
γ Glu-Ala	3.31 ± 0.25 eB	0.00 ± 0.00 eC	0.00 ± 0.00 eC	3.56 ± 0.36 eC	2.30 ± 0.35 eB	65.05 ± 1.99 dB	87.65 ± 5.38 cA	139.60 ± 4.86 aA	122.45 ± 6.53 bA	90.22 ± 3.91 cA	70.26 ± 1.14 dB	82.56 ± 4.68 cB
γ Glu-Val	2.14 ± 0.14 gB	272.56 ± 4.97 eB	549.80 ± 24.42 cB	296.28 ± 0.26 eC	3.09 ± 0.38 gB	434.00 ± 4.71 dA	691.61 ± 44.03 aA	644.11 ± 12.56 abA	95.42 ± 3.69 fA	454.07 ± 20.50 dA	634.07 ± 0.31 bA	527.67 ± 4.98 cB
γ Glu-Phe	2.17 ± 0.31 fB	2.45 ± 0.34 fC	3.30 ± 0.55 fC	3.10 ± 0.20 fC	3.59 ± 0.43 fB	203.52 ± 2.84 cA	292.21 ± 14.86 bA	356.00 ± 6.10 aA	111.17 ± 4.81 eA	195.25 ± 4.93 cB	163.49 ± 1.88 dB	150.96 ± 4.48 dB
γ Glu-His	7.39 ± 0.42 eB	4.16 ± 0.54 eB	4.64 ± 0.14 eC	3.17 ± 0.04 eC	3.33 ± 0.37 eB	63.53 ± 2.08 cdA	102.84 ± 5.76 bA	134.82 ± 0.79 aA	70.84 ± 3.71 cA	64.15 ± 3.44 cdA	57.45 ± 1.29 dB	58.99 ± 4.52 dB
γ Glu-Gln	2.48 ± 0.22 dB	0.00 ± 0.00 dC	0.00 ± 0.00 dC	0.00 ± 0.00 dC	0.00 ± 0.00 dB	19.84 ± 0.76 dB	46.34 ± 3.24 dB	67.66 ± 1.35 dB	1397.67 ± 98.69 aA	477.72 ± 48.04 bA	275.57 ± 5.10 cA	267.97 ± 9.84 cA
γ Glu-Gly	7.13 ± 0.44 eB	3.59 ± 0.15 eC	2.95 ± 0.39 eC	0.00 ± 0.00 eC	13.71 ± 0.35 eB	81.76 ± 2.35 dB	110.76 ± 6.97 cdA	238.30 ± 7.41 bA	552.84 ± 34.81 aA	135.48 ± 21.76 cA	89.43 ± 2.16 dB	135.11 ± 8.33 cB
γ Glu-Leu	3.69 ± 0.25 gB	0.00 ± 0.00 gC	0.00 ± 0.00 gC	0.00 ± 0.00 gC	4.56 ± 0.17 gB	237.61 ± 4.62 cA	324.24 ± 14.66 bA	408.24 ± 9.29 aA	134.79 ± 5.72 fA	200.72 ± 7.87 dB	167.75 ± 2.19 eB	157.24 ± 6.27 eB
γ Glu-Ile	0.00 ± 0.00 eB	0.00 ± 0.00 eC	0.00 ± 0.00 eC	0.00 ± 0.00 eC	0.00 ± 0.00 eB	80.15 ± 3.22 cA	130.04 ± 8.69 bA	168.26 ± 5.78 aA	65.63 ± 1.62 dA	70.33 ± 3.56 cdB	62.79 ± 5.16 dB	62.33 ± 2.68 dB
γ Glu-Met	2.25 ± 0.09 eB	0.00 ± 0.00 eC	0.00 ± 0.00 eC	0.00 ± 0.00 eC	2.03 ± 0.19 eB	87.68 ± 2.37 cB	107.25 ± 10.80 bA	135.60 ± 7.76 aA	67.01 ± 3.81 dA	102.03 ± 8.63 bcA	64.28 ± 5.57 dB	60.56 ± 2.87 dB
γ Glu-Tyr	2.07 ± 0.28 gB	39.84 ± 0.18 fB	40.47 ± 0.50 fC	32.16 ± 0.81 fC	2.65 ± 0.39 gB	154.19 ± 4.47 cA	185.50 ± 12.66 bA	251.67 ± 2.33 aA	81.38 ± 3.84 eA	147.48 ± 4.65 cA	113.06 ± 2.19 dB	118.25 ± 5.33 dB
**γ Glutamyl dipeptides**	**47.15 ± 5.33 hB**	**332.73 ± 5.32 gC**	**611.78 ± 25.22 fB**	**346.69 ± 0.95 hC**	**46.19 ± 2.50 hB**	**1522.29 ± 31.05 eB**	**2200.03 ± 132.12 cA**	**2741.56 ± 51.34 bA**	**3213.15 ± 129.02 aA**	**2073.30 ± 126.89 cA**	**1790.90 ± 37.68 cdA**	**1730.93 ± 47.19 dB**
γ Glu-Val-Gly	1.81 ± 0.09 efB	9.43 ± 0.57 dA	17.44 ± 1.11 aA	12.41 ± 0.78 cA	2.93 ± 0.46 eA	8.84 ± 0.02 dA	14.05 ± 1.19 bcB	15.27 ± 1.57 abA	0.00 ± 0.00 fC	9.51 ± 1.39 dA	14.16 ± 1.43 bcB	15.19 ± 0.98 abcA
γ Glu-Cys-Gly	492.77 ± 25.67 bcA	505.36 ± 16.69 abA	538.76 ± 23.80 aA	433.26 ± 10.29 dA	226.10 ± 3.32 eB	236.23 ± 4.96 eC	252.41 ± 5.37 eC	248.28 ± 7.17 eB	232.60 ± 3.60 eB	447.95 ± 13.44 cdB	449.04 ± 19.50 cdB	441.80 ± 23.88 dA
**γ Glutamyl tripeptides**	**494.58 ± 25.58 bcA**	**514.80 ± 16.15 abA**	**556.20 ± 22.89 aA**	**445.87 ± 9.93 dA**	**229.02 ± 2.89 eB**	**245.06 ± 4.95 eC**	**266.45 ± 4.27 eC**	**263.54 ± 5.90 eB**	**232.60 ± 3.60 eB**	**457.46 ± 12.06 cdB**	**463.20 ± 18.08 cdB**	**456.99 ± 24.83 cdA**
α Glu-Glu	0.00 ± 0.00 gB	51.24 ± 4.52 fB	219.47 ± 16.67 bA	67.77 ± 3.13 efB	0.00 ± 0.00 gB	73.01 ± 1.05 defA	206.57 ± 12.93 bA	91.03 ± 7.17 cdA	413.50 ± 1.00 aA	73.91 ± 7.77 deA	208.91 ± 4.14 bA	105.68 ± 7.90 cA
**Koku-related peptides**	**541.73 ± 20.49 hB**	**992.29 ± 7.99 gC**	**1337.74 ± 51.90 fB**	**883.67 ± 10.88 gC**	**275.08 ± 2.68 iC**	**1948.71 ± 36.61 eB**	**2642.19 ± 141.30 cdA**	**3139.63 ± 47.84 abA**	**3446.70 ± 130.17 aA**	**2706.18 ± 130.58 bcA**	**2433.74 ± 24.71 cdA**	**2325.77 ± 75.35 dB**

^a^ C, control or unhydrolyzed sample; A, Alcalase 4.0L Pure hydrolyzate; F, Flavourzyme1000L hydrolyzate; PP, Protana Prime hydrolyzate; U, Protana Uboost post-treatment; G, addition of free glutamine at final concentration of 20 mmol/L, 0, no treatment. ^b^ Lowercase different letters (a, b, c, d, e, f, g, and h) represents significant differences (*p* < 0.05) among all samples, while uppercase different letters (A, B, and C) reflect significant differences (*p* < 0.05) within the same group (unhydrolyzed samples: HC, HCU, and HCUG; single Alcalase hydrolyzates: HA, HAU, and HAUG; sequential Alcalase Flavourzyme hydrolyzates: HAF, HAFU, and HAFUG; and sequential Alcalase Protana Prime hydrolyzates: HAPP, HAPPU, and HAPPUG).

## Data Availability

The raw data supporting the conclusions of this article will be made available by the authors on request.

## Supplementary Materials

The following supporting information can be downloaded at: https://www.mdpi.com/article/10.3390/ijms27083440/s1.
