# Supplementary material for: Generation of Koku-Related Peptides Using Gamma-Glutamyl Transpeptidase Post-Treatment in Porcine Liver Hydrolyzates
Source: Int J Mol Sci. 2026 Apr 11;27(8):3440. doi: 10.3390/ijms27083440 (PMC13115641; doi:10.3390/ijms27083440)
Supplement: Supplementary file 1 [file ijms-27-03440-s001.zip › ijms-4208956-supplementary.pdf]

SUPPLEMENTARY MATERIAL

Table S1. Compilation of standard peptide properties for their analysis in HPLC-MS/MS.

Peptides	Retention time (min)	Precursor ion[b] (m/z; [M + H⁺]+AQC)	Standard curve	R^2
Leu-Glu	14.36	431.1920	Y = -0.000115565+0.011069*X	0.9968
Leu-Ala	14.26	373.1865	Y = -8.91798e-005+0.0127179*X	0.9938
γ Glu-Glu	7.83	447.1508	Y = -1.112e-005+0.00368364*X	0.9973
γ Glu-Ala	8.58	389.1454	Y = -4.01386e-006+0.00680442*X	0.9969
γ Glu-Val	11.78	417.1763	Y = -8.1999e-005+0.00871286*X	0.9968
γ Glu-Phe	14.95	465.1763	Y = -4.32789e-005+0.00629973*X	0.9974
γ Glu-His	5.85	228.0870	Y = -2.66491e-005+0.00400888*X	0.9983
γ Glu-Gln	6.99	446.1665	Y = -6.74405e-005+0.00580542*X	0.9958
γ Glu-Gly	7.56	375.1294	Y = -6.99775e-005+0.00883145*X	0.9986
γ Glu-Leu	14.14	431.1920	Y = -6.99775e-005+0.00883145*X[c]	0.9987
γ Glu-Ile	13.87	431.1920	Y = -2.98826e-005+0.00314123*X	0.9972
γ Glu-Met	11.63	449.1484	Y = -4.92672e-005+0.00493238*X	0.9908
γ Glu-Tyr	11.25	481.1712	Y = -5.01908e-005+0.00384567*X	0.9984
γ Glu-Val-Gly	10.59	474.1977	Y = -0.000960412+0.0987691*X	0.9972
γ Glu-Cys-Gly	*2.65*[a]	308.0909	Y = -3.25929e+006+1.06157e+006*X	0.9947
α Glu-Glu	7.97	447.1508	Y = -7.15915e-005+0.00423301*X	0.9971

[a] γ Glu-Cys-Gly was measured without derivatization. [b] The precursor ion for all the peptides was (m/z; [M + H⁺]+AQC), except for γ Glu-His (m/z; ([M + H⁺]+AQC) /2) , since it has two positive charges, and γ Glu-Cys-Gly (m/z; [M + H⁺]), since it was not derivatized. All precursor ions were sourced in the University of Washington's Proteomics Resource (UWPR) using the peptide fragmentation tool, (https://proteomicsresource.washington.edu/cgi-bin/fragment.cgi) (Accessed on 18/10/2025). [c] Standard curve for γ Glu-Ile was the same as γ Glu-Leu, due to not having the standard for this peptide.

Table S2. Pearson correlation values of antioxidant activities and hydrolysis parameters.

	OPA	ABTS	DPPH	FRAP	ORAC	Fe^{2+}CP	Protana Uboost
ABTS	0.975						
DPPH	0.664	0.659					
FRAP	0.926	0.889	0.546				
ORAC	0.974	0.973	0.627	0.881			
Fe^{2+}CP	0.602	0.626	-0.051	0.709	0.622		
Protana Uboost	0.068	0.057	-0.571	0.234	0.090	0.782	
Gln 20mM	0.065	0.050	-0.337	0.232	0.088	0.509	0.500
